# Evaluation and Characterization of Tamarind Gum Polysaccharide: The Biopolymer

**DOI:** 10.3390/polym13183023

**Published:** 2021-09-07

**Authors:** Rishabha Malviya, Sonali Sundram, Shivkanya Fuloria, Vetriselvan Subramaniyan, Kathiresan V. Sathasivam, Abul Kalam Azad, Mahendran Sekar, Darnal Hari Kumar, Srikumar Chakravarthi, Omji Porwal, Dhanalekshmi Unnikrishnan Meenakshi, Neeraj Kumar Fuloria

**Affiliations:** 1 Department of Pharmacy, School of Medical & Allied Sciences, Galgotias University, Greater Noida 201310, Uttar Pradesh, India; rishabha.malviya@galgotiasuniversity.edu.in (R.M.); sonali.sundram@galgotiasuniversity.edu.in (S.S.); 2 Faculty of Pharmacy, Centre of Excellence for Biomaterials Engineering, AIMST University, Bedong 08100, Kedah, Malaysia; 3 Faculty of Medicine, Bioscience and Nursing, MAHSA University, Jalan SP 2, Bandar Saujana Putra, Jenjarom 42610, Selangor, Malaysia; drvetriselvan@mahsa.edu.my (V.S.); srikumar@mahsa.edu.my (S.C.); 4 Faculty of Applied Science, Centre of Excellence for Biomaterials Engineering, AIMST University, Bedong 08100, Kedah, Malaysia; skathir@aimst.edu.my; 5 Advanced Drug Delivery Laboratory, Faculty of Pharmacy, International Islamic University Malaysia, Kuantan 25200, Pahang Darul Makmur, Malaysia; azad2011iium@gmail.com; 6 Department of Pharmaceutical Chemistry, Faculty of Pharmacy and Health Sciences, Universiti Kuala Lumpur Royal College of Medicine Perak, Ipoh 30450, Perak, Malaysia; mahendransekar@unikl.edu.my; 7 Jeffrey Cheah School of Medicine & Health Sciences, Monash University, No.3 Jalan Masjid Abu Bakar, 80100 Johor Bahru, Johor, Malaysia; hari.kumar@monash.edu; 8 Department of Pharmacognosy, Tishk International University, Erbil 44001, Iraq; omji.porwal@tiu.edu.iq; 9 College of Pharmacy, National University of Science and Technology, Muscat 130, Oman; dhanalekshmi@nu.edu.om

**Keywords:** tamarind gum, polysaccharides, micromeritic properties, pharmaceutical excipients

## Abstract

Polymers from natural sources are widely used as excipients in the formulation of pharmaceutical dosage forms. The objective of this study was to extract and further characterize the tamarind gum polysaccharide (TGP) obtained from *Tamarindus indica* as an excipient for biomedical applications. Double distilled water was used as a solvent for the extraction of gum while Ethyl alcohol was used as an antisolvent for the precipitation. The results of the Hausner ratio, Carr’s index and angle of repose were found to be 0.94, 6.25, and 0.14, respectively, which revealed that the powder is free-flowing with good flowability. The gum was investigated for purity by carrying out chemical tests for different phytochemical constituents and only carbohydrates were found to be present. The swelling index was found to be 87 ± 1%, which shows that TGP has good water intake capacity. The pH of the 1% gum solution was found to be neutral, approximately 6.70 ± 0.01. The ash values such as total ash, sulphated ash, acid insoluble ash, and water-soluble ash were found to be 14.00 ± 1.00%, 13.00 ± 0.05%, 14.04 ± 0.57% and 7.29 ± 0.06%, respectively. The IR spectra confirmed the presence of alcohol, amines, ketones, anhydrides groups. The contact angle was <90°, indicating favorable wetting and good spreading of liquid over the surface The scanning electron micrograph (SEM) revealed that the particle is spherical in shape and irregular. DSC analysis shows a sharp exothermic peak at 350 °C that shows its crystalline nature. The results of the evaluated properties showed that TGP has acceptable properties and can be used as a excipient to formulate dosage forms for biomedical applications.

## 1. Introduction

Natural polymers usually have unique properties that distinguish them from synthetic polymers, and tamarind gum polysaccharide (TGP) is one of them. TGP has a wide range of beneficial properties that makes it a suitable excipient for various applications [1]. Tamarind is a large evergreen tree that grows abundantly in the dry regions of Central and South India, as well as other Southeast Asian nations [2]. Plant-derived polysaccharides have piqued interest due to their wide range of pharmaceutical applications, including diluents, binder, and disintegrants in tablet formulation, thickeners in oral liquids, protective colloids in suspensions, gelling agents in gels, and bases in suppository formulation.

**Figure 1:**
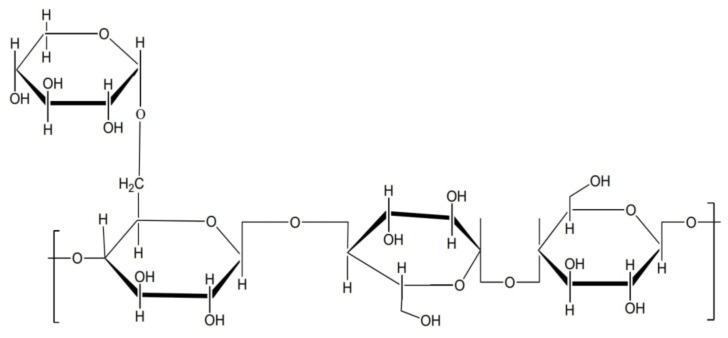
Chemical structure of tamarind seed polymer.

TGP is a plant polysaccharide derived from the Tamarindus indica Linn seed endosperm from Fabaceae family. TGP have 1735 kDa molecular weight [3]. It is a water-soluble, nonionic, branched polysaccharide with hydrophilic, gel-forming, and mucoadhesive characteristics [4]. TGP is also biodegradable, biocompatible, noncarcinogenic, and irritant-free. It is used in the pharmaceutical, cosmetic, and food industries as a promising biopolymer [5]. In recent years, it has been extensively studied and used as a successful pharmaceutical excipient in a variety of drug delivery applications. Tamarind gum is used in the development of drug delivery systems for the oral, intestinal, ophthalmic, buccal, and nasal routes [6]. The chemical structure of tamarind seed polymer are shown in Figure 1.

Gums, also known as polysaccharides, are water-soluble complex carbohydrates that may be used to make gels and mucilages. Galactose, arabinose, rhamnose, xylose, and galacturonic acid are only a few of the sugars that may be used to make them [7]. They have gelling, thickening, moisture retention, emulsification, and stabilizing properties. Polysaccharides are frequently utilized in food product manufacturing due to their versatility [8]. Its chemical structures are closely connected to its wide range of uses. The characteristics of polysaccharide or glycoconjugate can be determined by analyzing structural molecules properties [9].

The seed of the tamarind contains around 65% of the gum and might be utilized to develop specific drug delivery systems. The polysaccharide that is present in TGP is known as tamarind seed polysaccharide [10]. The physicochemical characteristics of TGP may be improved by converting it to carboxymethyl tamarind gum. TGP has certain important limitations, besides its suitability for therapeutic uses. The color of TGP is dull, and it has a characteristics odor [11]. *T. indica* extracts were found to exhibit antibacterial, antifungal, hypoglycaemic, cholesterolemic, cytotoxic, anti-inflammatory, gastrointestinal, hypolipomic, and antioxidant properties in pharmacological studies. The phytochemical analysis of a methanolic extract of *T. indica* leaves yielded two triterpenes, lupanone and lupeol. Scientists were prompted to chemically alter its functional groups due to its insolubility in water and deterioration in an aquatic environment [12]. Various modifications which have been executed till date include carboxymethylation, acetylation, hydroxyl-alkylation and thiolisation, polymer grafting [13].

The solubility, viscosity, swelling, and stability of tamarind gum have all changed as a result of these changes. TGP and CMTG have been employed to develop a variety of drug delivery systems [14]. Accordingly, there is a need to investigate TG’s physicochemical characteristics to identify its suitability as an excipient in the development of drug delivery systems. As a result, research was conducted to extract, modify, and characterize tamarind gum [15]. TGP is a highly viscous, mucoadhesive, and biocompatible natural polymer; it is also suitable for ocular drug delivery and preparation of ophthalmic medicaments. It has been used as a polymer for the formulation of ophthalmic drug delivery. Because the eye is such a sensitive organ and an immune privileged region, the biocompatibility of the polymers plays an important role in ocular drug delivery systems [16,17,18].

Various functionally derivatized TGP has recently gained popularity as potential pharmaceutical excipients in a variety of improved drug delivery systems, owing to their improved stability (lower degradability) [19]. These functionally derivatized tamarind gums have improved mechanical behavior and are capable of regulating drug release over a longer period. The present study deals with a detailed evaluation of several types of tamarind gum functionalizations for application in the development of better drug delivery systems. The first section discusses the origins, components, characteristics, and applications of tamarind gum. The latter section provides a thorough examination of tamarind gum functionalizations in drug administration [19,20].

## 2. Materials and Methods

Ethyl alcohol (90% *v*/*v*) was purchased from Sigma Aldrich Pvt. Ltd., Bangalore, India. All other chemicals are procured from HiMedia Laboratories Pvt. Ltd., Mumbai, India. All the reagents are analytical grade and used as supplied.

### 2.1. Collection of Plant Material

Crude plant material (Tamarindus indica) was purchased from a local shop in Greater Noida, India. The collected plant material was identified by the Department of Biotechnology, Gautam Buddha University (State Govt. University) Greater Noida, Uttar Pradesh, India.

### 2.2. Extraction of Gum

Extraction of Tamarind Gum Polymer

The test of the seeds of tamarind was removed by drying it in a hot air oven for 20 min at 40 °C. Then the seed coat was removed by simply crushing from the aside. The obtained white portions of the seeds were then utilized for the isolation technique. The seeds were immersed in a beaker with double distilled water and provided heating conditions till 40 °C. After extraction the supernatant fluid was discarded after the centrifugation technique. Then, the remaining portion was concentrated by heating them again at a constant temperature of 40 °C. The whole slurry was filtered and precipitate using ethyl alcohol. Then the precipitated product was spread on the petri dish and dried and size was reduced till fine powder was obtained [20,21].

### 2.3. Physicochemical Characterization of Gum

All the following physicochemical charcaterizations were carried out at 25 ± 2 °C.

#### 2.3.1. Identification Tests for Carbohydrates, Proteins, Tannins

The aqueous solution of extracted gum was used for chemical characterization. Test for carbohydrates, proteins, alkaloids, lipids, tannins, glycosides were performed according to the standard procedure [22,23].

##### Test for Carbohydrates

One percent of the solution of TGP was prepared in distilled water. 2 drops of α-naphthol was added to the solution in the test tube. The test tube was inclined carefully and 1 mL concentrated sulfuric acid was poured dropwise. Violet color appeared at the junction of the two liquids [24].

##### Test for Proteins

One percent of the solution of TGP was prepared using double distilled water, Biuret reagent was added. The presence of red color indicates the absence of proteins [25].

##### Test for Glycosides

Pure TGP was dissolved in a mixture of 1% ferric sulphate solution in (5%) glacial acetic acid. Add one or two drops of concentrated sulfuric acid. The red color was obtained due to the presence of 2-deoxy [26].

##### Test for Tannins

In the native TGP, 5% ferric chloride was added and boiled. The absence of greenish precipitate was an indication of an absence of tannins.

##### Test for Alkaloids

One percent of the sample solution was prepared and Dragendorff’s reagent was added. No brick red precipitates were obtained. Glycoside was found to be absent.

##### Sudan Red–III Test

To 1% sample solution of TGP, Sudan red III was added. No change in the color of the solution detects the absence of lipids in the polymer [27].

#### 2.3.2. Organoleptic Properties

The gum was characterized for organoleptic properties such as; taste as color, odour and texture.

#### 2.3.3. pH of Gum

Firstly, the tamarind gum polysaccharide was weighed and then dissolved in water separately to get a 1% *w/v* solution. The pH of the solution was determined by using a digital pH meter (DECIBLE, Chandigarh, India). pH instrument was calibrated using standard pH solutions 7 and 4. For the preparation of the standard solution, buffer capsules of pH 7 and pH 4 were dissolved in 100 mL of double-distilled water. Further, the gum solution was poured into a beaker (50 mL) to determine the pH (25 ± 2 °C).

#### 2.3.4. Viscosity of Gum

TGP was dissolved in an aqueous solution and filtered. 1% *w/v* solution of TGP was prepared. A relative density bottle is used to determine the density of the polymer solution. To determine the thermodynamic parameter, calibrated laboratory scale thermometer was used (25 ± 2 °C). The time is noted in triplicate and then the viscosity is calculated. The viscosity of the prepared 1% *w/v* solution of gum (25 mL) was measured using Ostwald viscometer [28]. Initially, the density of the solution was determined using a relative density bottle. The solution was then filled in the Ostwald viscometer and time was noted. Further, the viscosity of the sample was calculated using Equation (1) [29].

(1) $$\frac{\eta 2}{\eta 1}=\frac{\sigma 2-t2}{\sigma 1-t1}$$

where η2 = viscosity of sample, η1 = viscosity of water, σ2 = density of sample, σ1 = density of water, t2 = time taken by the sample, and t2 = time taken by water.

#### 2.3.5. Surface Tension

The surface tension of prepared 1% *w/v* solution of gum (25 mL) was measured using a stalagmometer (25 ± 2 °C). Initially, the density of the solution was determined using a relative density bottle. The solution was then filled in the stalagmometer through capillary action and the time of the drop falling was noted. Further, the surface tension of the sample was calculated using Equation (2) [30].

(2) $$\frac{\gamma 2}{\;\gamma 1}=\frac{\eta 1\cdot \;\sigma 2}{\eta 2\cdot \sigma 1}$$

where γ2 = surface tension of the sample, γ1 =surface tension of sample, η2 = Viscosity of sample, η1 = Viscosity of water, σ2 = Density of sample, and σ1 = Density of water.

#### 2.3.6. Swelling Index

The swelling index of the tamarind gum polysaccharide was calculated by weighing a butter paper of size 2.2 cm. Further butter paper was dipped in a petridish contain 15 mL of water. 0.1 gm of the powdered sample was kept over butter paper placed in a petridish and the weight of swelled TGP was measured a after 24 h at 25 ± 2 °C, and the final swelling index was calculated by Equation (3) [31].

(3) $$\mathrm{Swelling}\;\mathrm{index}=\frac{\mathrm{Initial}\;\mathrm{weight}-\mathrm{final}\;\mathrm{weight}}{\mathrm{Initial}\;\mathrm{weight}}\times 100$$

#### 2.3.7. Bulk Density and Bulkiness

Inverse bulk density was known as bulkiness. For bulk density determination, an accurately weighed quantity of sample (5 gm) was placed in a measuring cylinder. The cylinder was fixed on the bulk density apparatus and the volume occupied by the powder was noted at 25 ± 2 °C. Bulk density was calculated in Equation (4).

(4) $$\mathrm{Bulk}\;\mathrm{Density}=\frac{\mathrm{Weight}\;\mathrm{of}\;\mathrm{powder}}{\mathrm{weight}\;\mathrm{of}\;\mathrm{apparent}\;\mathrm{volume}}$$

#### 2.3.8. Powder Compressibility (Carr’s Compressibility Index)

Finely powdered tamarind gum polysaccharide (5 g) was taken and transferred into measuring cylinder and calculations were done using bulk density apparatus. Finally, Carr’s index was calculated at 25 ± 2 °C using Equation (5).

(5) $$\mathrm{Car}\;r\text{′}s\;\mathrm{index}=\frac{\mathrm{Tapped}\;\mathrm{density}-\mathrm{bulk}\;\mathrm{density}}{\mathrm{tapped}\;\mathrm{density}}\times 100$$

#### 2.3.9. Powder Flow Property

Flow characteristics were measured by the angle of repose. TGP (5 g) was poured from a funnel which was kept at a distance of 5 cm from the surface. The TGP formed a heap of a pile on the surface from where the radius can be calculated. The angle of repose can be determined using Equation (6).

(6) tanθ=h/r

where, h = height of pile, and r = radius of pile

### 2.4. For Powder Blend

The randomly picked sample were analyzed using a microscope to determine the minor and major axis length. The size of the particles was determined [32,33].

#### 2.4.1. Aspect Ratio

Aspect ratio is a term that describes the ratio between the size of a geometric shape and different sizes of them. The aspect ratio varies between 0 and 1 with a less value indicative of an elongated particle and circle having an aspect ratio of 1. The aspect ratio is “the proportional relationship between a minimum and maximum diameter of the particles.

(7) $$\mathrm{Aspect}\;\mathrm{ratio}=\frac{b}{l}$$

where b = minimum diameter of the particles, and l = maximum diameter of the particles

##### Roundness

Roundness is a measure of how closely the shape of the powder? It is based on the ratio between inscribed and the circumscribed circles that is the minimum and maximum sizes of the particles. The powder resembles a circle a perfect circle has a roundness value of 1.

(8) $$\mathrm{Roundness}=\frac{4\times \pi \times A}{{\left(P\right)}^{2}}$$

where, A = projected area of the particles, and P = perimeter of the particles,

##### Irregularity (IR)

Irregularity measure the surface area as compared to the size of the particle.

(9) $$\mathrm{Irregularity}=\frac{P\;}{L}$$

where, P = perimeter of the particles and L = maximum diameter

##### Elongation Ratio

The elongation ratio can be determined by “the proportional relationship between the maximum and minimum diameter of the particles.

(10) $$\mathrm{Elongation}\;\mathrm{ratio}=\frac{l}{b}$$

where, l = Maximum length of the particle and b = Minimum length of the particle

##### Equivalent Circle Diameter (ECD)

Equivalent circle diameter can be determined by the diameter of the particle with the same cross-section area as the powder. The greater the equivalent circle diameter, the higher the mean particle size.

(11) $$\mathrm{Equivalent}\;\mathrm{circle}\;\mathrm{diameter}=\sqrt[{2}]{A/\pi }$$

where, A = projected area of the particle.

##### Hausner Ratio

It can be determined by the flowability of the powder. It is also used in industries as an indication of the flowability of the powder. A Hausner ratio is greater than 1.25, so it’s can be indicating poor flowability.

(12) $$\mathrm{Hausner}\;\mathrm{Ratio}=\frac{\mathrm{Tapped}\;\mathrm{Density}\;}{\mathrm{Bulk}\;\mathrm{Density}}$$

##### Angle of Internal Friction (AIF)

Angle of internal friction is determining the relationship between the porosity and the number of taps. The AIF is derived by determining the angle made between the straight line and the abscissa.

(13) $$K=\varepsilon^{2\;}N/\left(1-\varepsilon \right)$$

where, N = number of taps and ε = porosity.

##### Porosity (ε)

The porosity is based on the apparent density (bulk density) and the true density of the compacted powders.

(14) $$\varepsilon =1-\frac{\mathrm{Apparent}\;\mathrm{Density}}{\mathrm{True}\;\mathrm{Density}}$$

### 2.5. Ash Value Determination

#### 2.5.1. Total Ash Value

1 g of TGP was taken in a crucible and ignited at a temperature of 600–700 °C. After 5 min, the ash was removed and was weighed till constant weight is achieved [34].

#### 2.5.2. Sulphated Ash Value

The preheated crucible was taken in which 1 g of the TGP was added. The material was ignited and converted to ash. Slightly moisten the content with dilute sulphuric acid and gently heat it, until white fumes are evolved. Calculate the weight of sulphated ash with respect to the air-dried TGP [34].

#### 2.5.3. Water-Soluble Ash

Initially convert the native TGP into ash and boil it with 25 mL distilled water for 5 min. Wash it thoroughly and weigh it. Subtract the weight of insoluble matter from the weight of the ash. Calculate the percentage of water-soluble ashes present [34].

#### 2.5.4. Acid Insoluble Ash

Initially convert the native TGP into ash and boil it with 25 mL dilute hydrochloric acid for 5 min. Collect the matter in the crucible and wash it thoroughly with water. Weight the content and calculate the acid insoluble ash present [34].

### 2.6. Particle Size Analysis

Particle size was determined by using optical microscopy. In the sample preparation, a ~100 microliters sample was placed over a slide and cover with the help of a coverslip with gentle pressure to achieve a sample thickness of ~25 micrometers. Calibration of optical microscope was done by ocular and stage micrometre scale. After calibration, the least count of stage scale was determined and no. of particles (approx. 60 particles) lies in between scale was noted and calculated [35,36].

### 2.7. FT-IR Spectral Analysis

The FTIR spectral analysis of TGP was performed using an Alpha Bruker ATR FTIR spectrophotometer (Alpha, ECD-ATR; Bruker Peoria, IL, USA). The sample is prepared by filling the ATR’s analyser plate with dried powdered samples directly. The spectrums were recorded in transmittance mode with 66 scans and a resolution of 2 cm^−1^ from 4000 to 600 cm^−1^. The spectra obtained were recorded and analysed to determine the functional groups present in the polysaccharide [37]. 32 scans were done and averaged to achieve a satisfactory signal-to-noise ratio [38].

### 2.8. Contact Angle Determination

The measuring of contact angle is an essential factor in determining wetting ability. A Rame goniometer model 100-00-230 (Rame-Hart-Instrument Co., Succasunna, NJ, USA) was used to calculate the contact angle. The contact angle was determined using a Rame goniometer as a direct tool. An aqueous solution of samples (1% *w*/*v*) was produced and cast on a glass surface, then vacuum dried at 40 °C [37].

### 2.9. Scanning Electron Microscopy Analysis (SEM)

SEM is generally used to determine the morphology of samples. The scanning electron microscope (SEM) was used to examine the surface characteristics of TGP. Using a Zeiss EVO 18 analyzer (ZEISS Research Microscopy Solutions, Jena, Germany), the surface characteristics of polymers were examined [39].

### 2.10. Differential Scanning Calorimetric Analysis (DSC)

TGP’s thermal analysis was performed with a Shimadzu DSC-60 (Kyoto, Japan). The experiment was conducted at temperatures ranging from 0 to 300 °C under nitrogen environment (heating rate of 10 °C/min and nitrogen purging rate of 50 mL/min) [40]

### 2.11. Moisture Content

The moisture content of the powder was carried out by thermos gravimetric method using IR moisture balance [41].

## 3. Results and Discussion

Tamarindus indica was isolated using distilled water as a solvent system and ethyl alcohol as precipitating agent. Phytochemical investigation showed the presence of carbohydrates while reducing sugar, glucose, tannins, proteins, and polysaccharides were absent. Likewise, the result is found in the study conducted on Azadirachta Indicia and Acacia Nilotica Gum performed by Rishi et al. [42]. Results after the phytochemical test are summarized in Table 1.

### 3.1. Chemical Characterization of Isolated Gum

Different chemical tests were performed on the TGP [43,44]. From the above test, it was concluded that carbohydrate was present and protein, tannins, lipids and glycosides were absent in the native TGP.

### 3.2. Organoleptic Properties and pH

Organoleptic properties of the polymer were observed and found to be acceptable. The color of powdered gum was light brownish. The odour of the polymer was found to be characteristic. The fracture of all the batches was rough. The polymer was soluble in hot water, swell to form a gel in cold water and insoluble in methanol, ethanol, benzene and acetone. Studies conducted on Artocarpus integer gum performed by Farooq et al. and Malviya et al. have also shown the same result and hence support this result [45,46].

### 3.3. pH

The pH of TGP (1% solution) was found to be 6.70 ± 0.01. approx. A similar result has been observed in the study of almond gum conducted by Farooq et al. that found pH 5.25 ± 0.813 which is slightly acidic [47].

### 3.4. Viscosity

**Figure 2:**
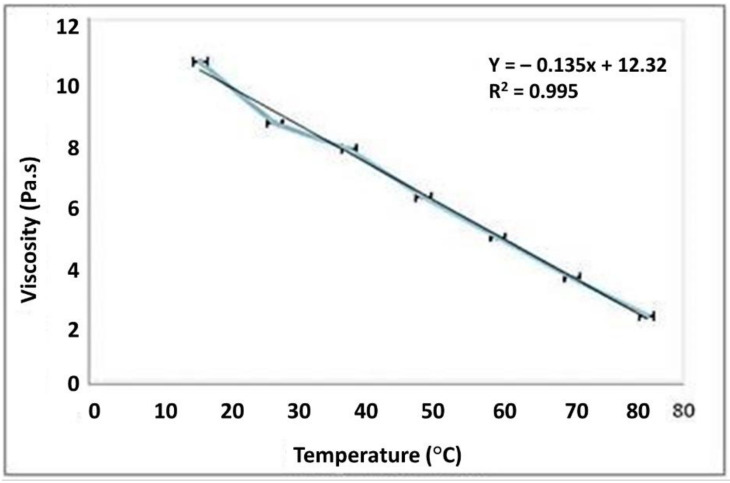
Graphical representation of temperature dependant viscosity (1% *w*/*v* solution of TGP).

The viscosity of fluid decreases with an increase in temperature. The viscosity of liquid depends upon the strength of the attractive forces between the molecules that directly depends on their composition, shape, size and kinetic energy of the molecules, which directly depend on the temperature [46]. This is because when the temperature of the system is raised; energy is added effectively which gives the molecules of the liquid the required energy to overcome the intermolecular force. The temperature dependant viscosity of TGP is shown in Figure 2.

### 3.5. Surface Tension

**Figure 3:**
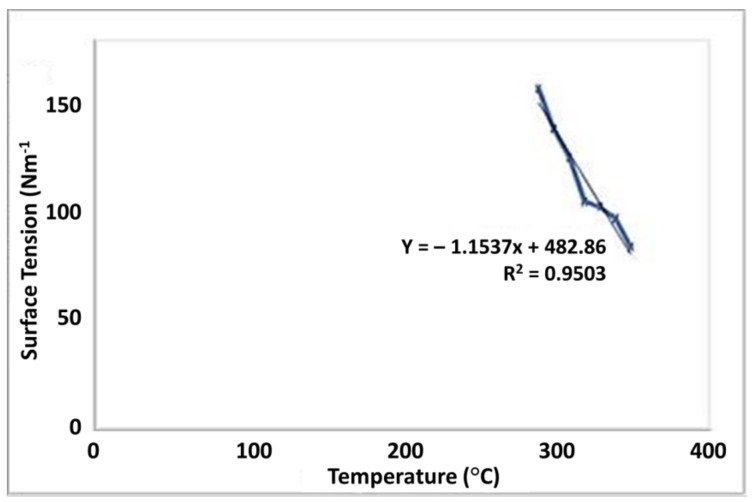
Graphical representation of temperature dependant surface tension.

Surface tension of the tamarind seed polymer decreases with an increase in temperature. With the rise in temperature, the kinetic energy of the molecules increases. Therefore, the strength of intermolecular forces decreases resulting in the decrease of surface tension [47]. Therefore, the strength of intermolecular forces decreases resulting in the decrease of surface tension. The temperature-dependent surface tension of TGP has shown in Figure 3.

### 3.6. Swelling Index

The swelling index was found to be 81.83%, which indicates that TGP has a good water intake capacity. This result was supported by Farooq et al. They also observed 87.44 ± 0.310% of the swelling index in their study [45]. As discussed in various studies swelling index has been determined for various polysaccharides, so in present investigation swelling index was used as a parameter for the characterization of gum.

### 3.7. Micromeritics Properties

The various physical and functional parameters of the gum are illustrated in Table 2. The results of the Hausner ratio, Carr’s index and angle of repose and density revealed that the powder is free-flowing with good flowability. These micromeritics properties of gum were supported by Aroshi Sharma et al. [48]. In a study by Aroshi et al., it was also observed that TGP had the Hausner ratio, Carr’s index and angle of repose of 1.25, 18 and 35°, respectively; hence, the obtained results from the present investigation are in accordance with previous studies.

### 3.8. Ash Value

According to Uzma Farroq et al., total ash, acid insoluble ash and water-soluble ash were found to be 15.9%, 0.57% and 3%, respectively; according to Deeksha et al. it was found to be 15.9%, 0.57% and 3%, respectively [43,49]. Likewise, the result was observed as described by authors elsewhere.

#### 3.8.1. Total Ash Values of TG

The ash value of TG was found to be 14.00 ± 1.00%. A similar result is observed in the study of Farooq et al. (total ash value 15.9%) [43]. This value shows that the TG has high inorganic content. The ash content was possibly due to the presence of Na^+^ and Ca^+^, which were not harmful. The observed ash value indicates that TG was richer in carbohydrates, and TG was served as a good source of dietary need. The total ash, acid insoluble ash and water-soluble ash were found to be 15.9%, 0.57% and 3%, respectively.

#### 3.8.2. Sulphated Ash Value

This determination was done to measure the number of residual substances which were not volatilized from TGP, when the sample was ignited in the presence of sulphuric acid. The sulphated ash value of TG was found to be 13.00 ± 0.05%. This value indicates that 13.00% of inorganic impurities were present in TG.

#### 3.8.3. Water-Soluble Ash Value

The water-soluble ash value predicts the nature and purity of crude drugs. The less extractive value, i.e., <5%, indicates that exhausted materials or adulterants were present in the crude drug. The water-soluble ash value was found to be 7.29 ± 0.06%. The obtained value showed that TG was pure and free from adulterants or exhausting materials.

#### 3.8.4. Acid-Insoluble Ash Value

The observed acid–insoluble ash value was found to be 14.04 ± 0.57%. This value showed that 14.04% of the proportion of the sample was not hydrolyzed by 72% of sulphuric acid and subsequently not volatilized upon the incineration of TG residue. All detail of found ash value has shown in Table 3.

### 3.9. Particle Size Analysis

The particle size of TG particles was found to be 88.54 ± 0.76 µm. It indicates that TG has coarse particle lies in-between its usual particle size range. The average size of 44.31 ± 9.43 µm was found in a study by Sumedha Pant et al., which supports this result [50]. The particle size distribution is shown in Table 4.

In the present study, the particle size of TG was found to be 88.54 ± 0.76 µm, while the organic material particle size which improves the mixed maricite’s efficiency and porosity of TG was found to be 0.197 ± 0.072 ε, which proves that TG has coarse particle which is satisfactory for the preparation of dosage form. The organic material of commercial carbon electrodes has a porosity of 0.76 cm^3^/g [51].

### 3.10. FT-IR Spectral Analysis

**Figure 4:**
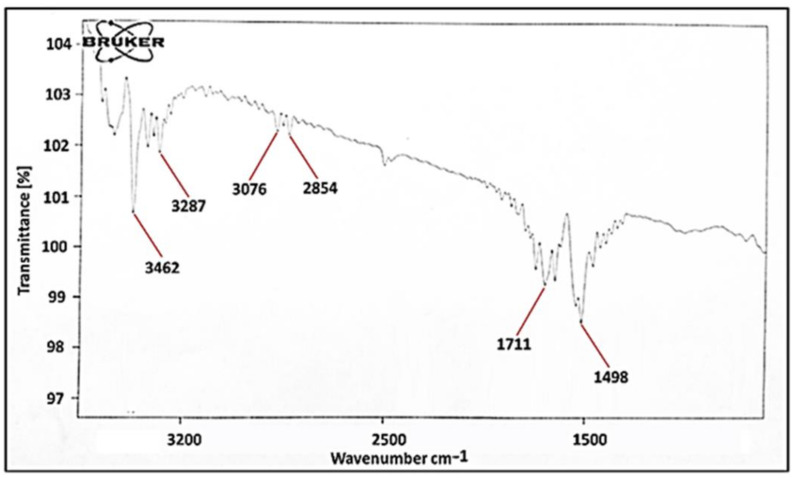
FTIR spectra of TGP.

According to a study by Nair et al., the FTIR spectrum of nebivolol depicts characteristic peaks at 3209.93 cm^−1^ (O–H stretching), 2873.42 cm^−1^ (C–H stretching), 1491.67 cm^−1^ (C=C stretching), 1349.93 cm^−1^ (C–N stretching), and 1141.67 cm^−1^ (C–O stretching) [52,53]. Based on the literary facts the presence of IR bands at 3462, 3287, 3076, 2854, 1711 and 1498 cm^−1^ in FTIR spectrum of TGP were assigned to O–H, N–H, C–H, C=O stretch and C=C functional groups of the TGP [54]. The result of the IR spectral study was also supported by the study performed by Kailas et al. and Chawananorasest et al. [55,56]. The native TGP does not contain any nitrogenous group in its structure. The main groups present in the TGP are carboxylic acid group (–COOH) and hydroxyl groups (–OH) (Figure 4). Due to this, the OH stretching is predominantly shown at 3416 cm^−1^ and C=O groups are highly dominating at about 1600–1800 cm^−1^ [57,58].

### 3.11. Contact Angle Determination

**Figure 5:**
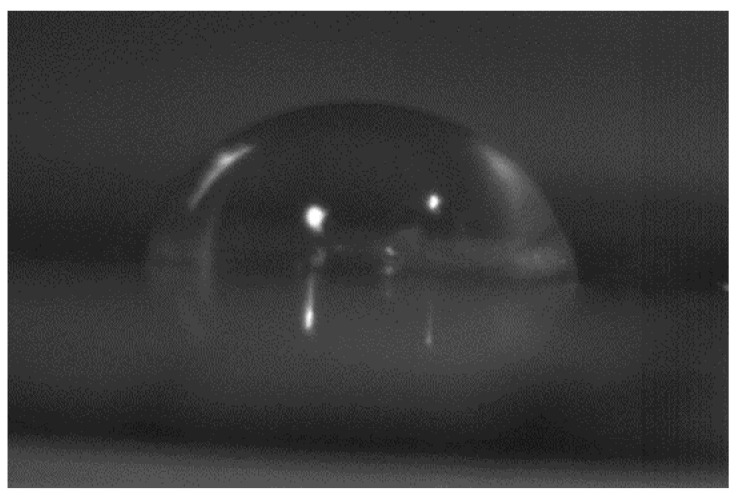
Contact angle measurement of TGP.

Copper plate was utilized for the drop formation. Further, the drop was vacuum dried and the plate was kept under the NYKON microlense from a distance of 22 cm (object piece). PHANTOM HIGH PEAK camera-1300 was utilized for the whole procedure. According to Malviya et al., the contact angle <90° indicates favorable wetting and good spreading of liquid over the surface; however, the contact angle > 90° indicates unfavorable surface wetting and formation of a compact droplet by liquid over a surface. The solubility of the polymer is inversely proportional to the contact angle. In the study of Malviya et al., NGP have a contact angle of 75.14 ± 2.61°, likewise the contact angle of the TGP was found to be 73.682 ± 2°, as described by authors elsewhere. A photograph image for the same is given in Figure 5 [59].

### 3.12. Scanning Electron Microscopy (SEM)

**Figure 6:**
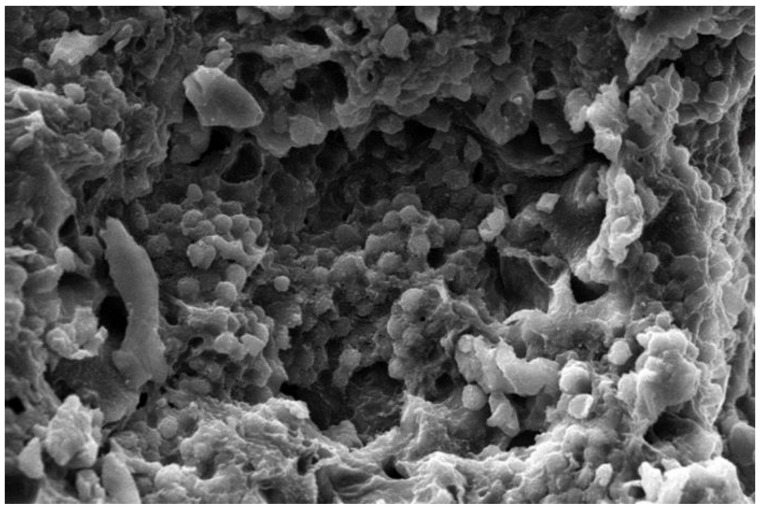
SEM image of TGP.

Mainly for the determination of the structural morphology of the native and modified sample, SEM is applied. Both the samples were gold coated to increase the conductivity of the electron beam passing them. The SEM of the TGP is shown in Figure 6. Likewise, in the result of the SEM analysis performed by Samrot et al. they observed that particles were spherical and that TGP powder has two types of particles, smaller sized particles with rough rounded edges and larger-sized particles with irregular shapes with smooth surfaces [60]. SEM results show that the TGP has sizes up to 30–90 µm. The SEM result was also supported by Malsawmtluangi et al., in which the investigator also observed smooth surfaces with some irregularities in particles [61].

### 3.13. Differential Scanning Calorimetric Analysis (DSC)

**Figure 7:**
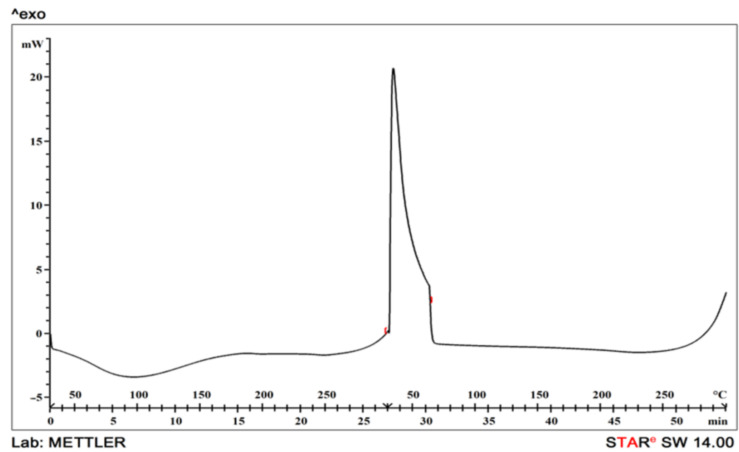
DSC thermogram of TGP.

The DSC of the native tamarind seed polymer was determined. The DSC of the pure and modified sample was in the heating range of 0–70 °C and heating rate of 10 ° per 10 min. The peak showing the melting point of the TGP was shown in Figure 7. The sharp endothermic peak at 229.31 °C was found in a study of Nair et al.; similarly, as described by the author elsewhere, in the case of TGP a sharp exothermic peak was evolved at 350 °C that shows its crystalline nature [52]. This study is also supported by Gaur et al., in which a sharp exothermic peak was found at 350 °C, which support its crystalline nature [62]. According to study of Malviya et al., a thermogram of NG–g–Am shows a single sharp exothermic peak at 249 °C. The sharp exothermic peaks of N1 show the crystalline nature of acrylamide graft copolymers that also supports its crystalline nature [63,64,65].

### 3.14. Moisture Content

The moisture content was found to be 8.2%, indicating that it has good stability for dosage from.

## 4. Biomedical Application of TGP

Due to multifunctional features, polysaccharide-based composites generated from natural sources have been widely exploited, particularly in drug delivery systems and biomedical applications. These materials are well-known and in high demand because of their biochemical capabilities, which are similar to those of animal cells and the human body. Natural polysaccharide-based nanomaterials may have a wide range of biological properties, including biocompatibility, bioavailability, and sustainability [66]. TGP is used as suspending and emulsifying agent in liquid oral dosage forms, binder in solid dosage form, novel controlled release modifiers, matrix oral drug release modifier, buccal drug release modifier, ophthalmic drug release modifier, carrier for colon targeted drug delivery [67,68,69].

## 5. Conclusions

TGP was extracted from the crude seed of Tamarindus indica and it was observed from research work that the gum of tamarind has carbohydrates. All the organoleptic properties evaluated were found to be acceptable. The color of powder was light brownish, odour was characteristic, fracture was rough. The pH was found to be almost neutral i.e., 6.70 ± 0.01. Surface tension of tamarind seed polymer decreases with an increase in temperature. Swelling index reveals that the gum swells well in water was found to be 87%. The bulk density was found to be 0.80 ± 0, tapped density was 2.55 ± 0.0071, carr’s index was 6.25 ± 0.0071, Hausner’s ratio was 0.94 ± 0.0071, angle of repose was 0.14 ± 0.1979, aspect ratio was 0.52 ± 0.071, roundness was 0.67 ± 0.073, irregularity was 3.05 ± 0.071, equivalent circle diameter was 3.75 ± 0.072, elongation ratio was 2.11 ± 0.074, degree of volume 0.053 ± 0.073, porosity was 0.197 ± 0.072, angle of internal friction was 1.88 ± 0.074 obtained. The total ash value was within the limits and found to be 14.00 ± 1.00, sulphated ash value was 13.00 ± 0.05, water-soluble ash was 7.29 ± 0.06 and acid-insoluble ash was 14.04 ± 0.57 obtained. The particle size of TGP particles was found to be satisfactory which indicates that TGP has coarse particles that are in-between its usual particle size range. The IR spectra confirmed the presence of alcohol, amines, ketones, anhydrides. The contact angle of the TGP was found out to be <90°, which indicates favorable wetting and good spreading of liquid over the surface. The SEM results showed a smooth surface with some irregularities in particles. The DSC of the sample was in the heating range of 0–70 °C and showed a sharp exothermic peak at 350 °C that displays its crystalline nature. Hence from the result, it was concluded that the evaluated properties showed that gum derived from tamarind seed has acceptable pH and organoleptic properties and can be used as a pharmaceutical excipient to formulate dosage forms. Extraction of gum from seed shows better management of waste material. So, extracted seed gum may be a suitable substitute for pre-established natural gums such as guar gum, gum acacia and starch etc.

## Figures and Tables

**Table 1:** Different chemical tests employed on TGP.

Test	Observation	Inference
Molish’s test	Purple ring at the junction of two liquid	Carbohydrate present
Benedict’s	Brick red precipitate	Carbohydrate confirmed
Osazone test	Needle shaped crystals	Confirmatory test for fructose and galactose
Biuret test	Red color	Protein absent
Test for tannins	No greenish precipitate obtained	Tannins absent
Sudan red III test	No change in color	Lipids absent
Dragendorff’s test	No red color obtained	Glycosides absent

**Table 2:** Micromeritics properties of TGP.

Property	Observation	Average
Bulk Density (g/cm^2^)	0.80 g/cm^2^	0.80 ± 0
Tapped Density (g/cm^2^)	0.83 g/cm^2^	2.55 ± 0.0071
	0.86 g/cm^2^	
	0.86 g/cm^2^	
Carr’s Index	3.75	6.25 ± 0.0071
	7.5	
	7.5	
Hausner’s Ratio	0.96	0.94 ± 0.0071
	0.93	
	0.93	
Angle of repose (*θ*)	0.14 θ	0.14 ± 0.1979
	0.13 θ	
	0.16 θ	
Aspect Ratio	0.5	0.52 ± 0.071
	0.75	
	0.33	
Roundness	0.69	0.67 ± 0.073
	0.76	
	0.58	
Irregularity	3	3.05 ± 0.071
	3.5	
	2.66	
Equivalent circle diameter	1.9	3.75 ± 0.072
	7.03	
	2.34	
Elongation Ratio	2	2.11 ± 0.074
	1.33	
	3	
	0.13	
Degree of volume	0.032	0.053 ± 0.073
	0.064	
	0.064	
Porosity (ε)	0.197 ε	0.197 ± 0.072
Angle of internal friction	0.946	1.88 ± 0.074
	1.892	
	2.83	

**Table 3:** Details of ash values of TG.

S.no	Parameters	Observation
1	Total ash value (%)	14.00 ± 1.00
2	Sulphated ash (%)	13.00 ± 0.05
3	Water-soluble ash (%)	7.29 ± 0.06
5	Acid-insoluble ash (%)	14.04 ± 0.57

**Table 4:** Particle size analysis of TG.

Size Range (µm)	No. of Particles
0–20	30
20–40	67
40–80	114
80–90	90
>90	40

## Data Availability

The data presented in this study are available on request from the corresponding author.
